# EGFR-targeting peptide-coupled platinum(IV) complexes

**DOI:** 10.1007/s00775-017-1450-7

**Published:** 2017-04-12

**Authors:** Josef Mayr, Sonja Hager, Bettina Koblmüller, Matthias H. M. Klose, Katharina Holste, Britta Fischer, Karla Pelivan, Walter Berger, Petra Heffeter, Christian R. Kowol, Bernhard K. Keppler

**Affiliations:** 1 Institute of Inorganic Chemistry, University of Vienna, Waehringer Strasse 42, A-1090 Vienna, Austria; 2 Institute of Cancer Research and Comprehensive Cancer Center, Medical University of Vienna, Borschkegasse 8a, A-1090 Vienna, Austria; 3 Research Cluster ‘‘Translational Cancer Therapy Research’’, University of Vienna, Waehringer Strasse 42, A-1090 Vienna, Austria

**Keywords:** Platinum complexes, Anticancer drug, Peptides, EGFR

## Abstract

The high mortality rate of lung cancer patients and the frequent occurrence of side effects during cancer therapy demonstrate the need for more selective and targeted drugs. An important and well-established target for lung cancer treatment is the occasionally mutated epidermal growth factor receptor (EGFR). As platinum(II) drugs are still the most important therapeutics against lung cancer, we synthesized in this study the first platinum(IV) complexes coupled to the EGFR-targeting peptide LARLLT (and the shuffled RTALLL as reference). Notably, HPLC–MS measurements revealed two different peaks with the same molecular mass, which turned out to be a transcyclization reaction in the linker between maleimide and the coupled cysteine moiety. With regard to the EGFR specificity, subsequent biological investigations (3-day viability, 14-day clonogenic assays and platinum uptake) on four different cell lines with different verified EGFR expression levels were performed. Unexpectedly, the results showed neither an enhanced activity nor an EGFR expression-dependent uptake of our new compounds. Consequently, fluorophore-coupled peptides were synthesized to re-evaluate the targeting ability of LARLLT itself. However, also with these molecules, flow cytometry measurements showed no correlation of drug uptake with the EGFR expression levels. Taken together, we successfully synthesized the first platinum(IV) complexes coupled to an EGFR-targeting peptide; however, the biological investigations revealed that LARLLT is not an appropriate peptide for enhancing the specific uptake of small-molecule drugs into EGFR-overexpressing cancer cells.

**Electronic supplementary material:**

The online version of this article (doi:10.1007/s00775-017-1450-7) contains supplementary material, which is available to authorized users.

## Introduction

Cancer is still a major cause of death with 8.2 million cases worldwide in 2012 [1, 2] and nearly 20% thereof due to lung cancer [3]. Despite being the most prominent cancer type, lung cancer is usually still a death warrant, not only because of the common late-stage diagnosis but also due to the bad response rate to chemotherapy [4]. Thus, at diagnosis, most patients are in stage III or stage IV with already established metastasis into bones, brain, adrenal gland and/or liver [5]. Although platinum-based drugs are often considered “old-fashioned”, these compounds are still of high importance for most treatment regimen in lung cancer therapy [6, 7]. Regardless, the overall response is quite low (around 20%) and—despite several dose and combination studies—the 1-year survival rate has only increased to around 25–30% during the last decades [8]. The reason for this unsatisfactory performance is that treatment has to be frequently discontinued due to drug resistance development [9] or severe side effects like nephrotoxicity, neurotoxicity and ototoxicity for cisplatin or myelosuppression for carboplatin [10]. This is based on the low tumor selectivity of the highly cytotoxic platinum(II) drugs resulting also in damage of healthy dividing tissue. To reduce the treatment-associated effects, the research is focusing on platinum(IV) prodrugs. Such complexes are less cytotoxic and are assumed to be reduced preferably inside the tumor tissue, in which they release the highly active platinum(II) compounds [11]. However, despite vigorous research, so far, no platinum(IV) compound (with satraplatin being the most prominent representative) was approved for clinical application [12, 13].

Nowadays, there are two main approaches to further optimize the specificity of chemotherapeutic drugs. One possibility is to attach or enclose the compound into macromolecular structures like nanoparticles, polymers, micelles, etc., and, thereby, accumulate the drug inside the tumor tissue due to the enhanced permeability and retention (EPR) effect, also known as passive targeting [14]. Secondly, a cancerous tissue can also be targeted actively by aiming for tumor-specific characteristics, such as overexpressed receptors or an altered metabolism [15, 16]. An example is the epidermal growth factor receptor (EGFR), which is overexpressed in several tumor types including breast, colon and non-small cell lung cancer (NSCLC) [17]. The importance of the EGFR signaling pathway already led to the approval of several therapeutics, which are based either on monoclonal antibodies, such as cetuximab and panitumumab, or on small-molecule inhibitors such as gefitinib, erlotinib or afatinib, especially in the treatment of EGFR-mutated NSCLC [17, 18]. A different approach of EGFR targeting is the use of EGFR-binding peptides. Generally, there are several peptide sequences, which have been reported in the literature to specifically bind the EGFR and, therefore, enhance drug uptake via receptor-mediated endocytosis. The most prominent peptide sequences are CMYIEALDKYAC [19, 20], YHWYGYTPQNVI (also known as “GE11”) [21–24] and LARLLT (also known as “D4”) [25–28]. For some highly cytotoxic drugs such as paclitaxel [23, 26] and doxorubicin [19, 20, 24], conjugates or targeted nanoformulations with these peptides were already reported to enhance the tumor specificity. Notably, such an approach has not been applied to platinum compounds so far. This is surprising as in the treatment of lung cancer both, the EGFR (as a target) as well as platinum-based anticancer drugs, are of high importance.

Therefore, the aim of this study was the development of the first EGFR-targeted platinum(IV) compounds functionalized with the EGFR-binding peptide LARLLT and to study their targeting effects. In course of this study, maleimide-containing platinum(IV) precursors based on cisplatin and oxaliplatin were prepared and coupled via a cysteine moiety to LARLLT or the shuffled sequence RTALLL, which was used as a reference compound for all experiments. Notably, chemical analyses revealed the occurrence of a transcyclization reaction between the coupling moieties maleimide and cysteine which, however, does not affect the targeting peptide sequence. Subsequent biological analyses of the new peptide-coupled platinum(VI) compounds showed, unexpectedly, no significant correlation with the EGFR status of the cancer cell lines. Therefore, fluorophore-coupled LARLLT and RTALLL were synthesized to re-evaluate the targeting ability of the peptide sequence itself. However, also these investigations did not reveal any EGFR-specific drug uptake in cell culture experiments. Consequently, we conclude in this study that LARLLT is not a suitable peptide for the EGFR-specific targeting of small-molecule drugs.

## Results and discussion

### Synthesis

**Scheme 1:**
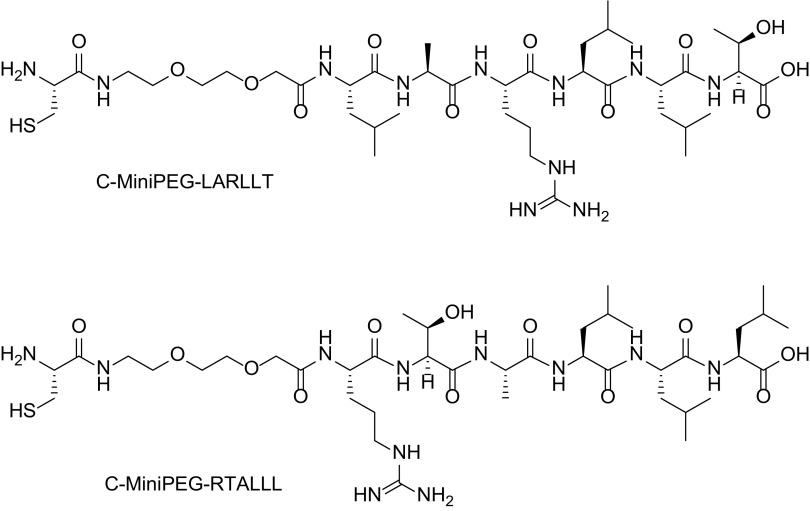
Structures of the cysteine-functionalized peptides C-MiniPEG-LARLLT and the shuffled reference C-MiniPEG-RTALLL

For all synthesized platinum compounds potassium tetrachloridoplatinate was used as a starting material. The respective platinum(II) cores, cisplatin [29] and oxaliplatin [30], were prepared according to standard, literature-known procedures. Subsequent oxidation with hydrogen peroxide was carried out either in methanol [31] or acetic acid [32] to yield the hydroxidomethoxidoplatinum(IV) species or the acetatohydroxidoplatinum(IV) species, respectively. The maleimide-functionalized platinum precursors **1** and **2** were synthesized in absolute DMF under inert conditions with subsequent chromatographic purification, as published recently [33]. Additionally, a succinimide-functionalized oxaliplatin compound **2**_**ref**_ was prepared analogously, which was used as the platinum(IV) reference compound. The cysteine-functionalized peptide C-MiniPEG-LARLLT (MiniPEG = 2-(2-(2-aminoethoxy)ethoxy)acetic acid) and the shuffled, reference sequence C-MiniPEG-RTALLL (see Scheme 1) were customer synthesized (trifluoroacetic acid (TFA) salts; >95% purity checked by elemental analysis; the amino acid sequence was verified by two-dimensional NMR spectroscopy).

**Scheme 2:**
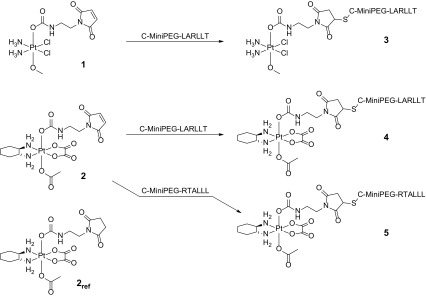
Reaction scheme for the synthesis of the EGFR peptide-coupled platinum(IV) complexes **3**–**5** out of compounds **1** and **2** and the structure of the platinum(IV) succinimide reference compound **2**_**ref**_

The MiniPEG linker was introduced to ensure a sufficient distance between the targeting peptide LARLLT/RTALLL and the platinum complex, whereas the cysteine moiety was used as a highly reactive moiety toward the maleimide-functionalized platinum(IV) precursors (see Scheme 2). As a first attempt, the coupling reactions were performed with an excess of the platinum complex **1** (which can be easily removed by preparative HPLC) and C-MiniPEG-LARLLT in buffered aqueous solution and monitored by RP-HPLC. Already the first measurement after mixing of the starting materials (after approximately 5 min) revealed a very fast binding of the peptide to the platinum(IV) precursor with formation of **3**.

However, after several hours of the coupling reaction in phosphate buffer at pH 7.4 the product peak (**3A**) strongly decreased and another peak (**3B**) with a higher retention time on the reversed-phase column was found. Surprisingly, mass spectrometry measurements showed that both peaks have exactly the same molecular mass. To investigate the reason for the difference in retention time and whether it is an intramolecular rearrangement or just a pH-dependent protonation/deprotonation, both product fractions **3A** and **3B** were purified and analyzed.

### Detailed investigations of the conversion reaction

**Fig. 1:**
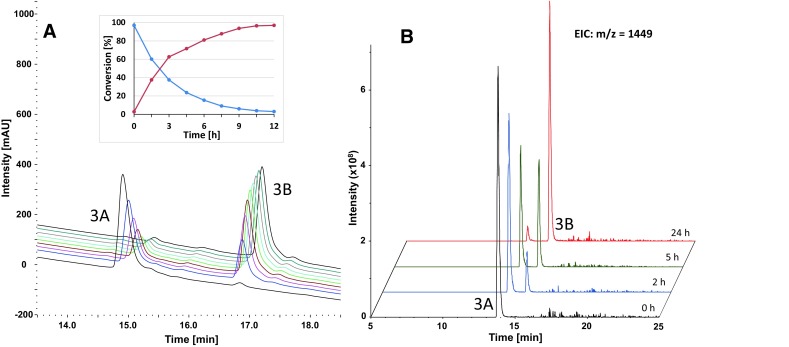
**a** Conversion of the primary product peak **3A** into the secondary product peak **3B** in 50 mM phosphate buffer (pH 7.4) over 12 h monitored by RP-HPLC at 210 nm (**b**) and by LC–MS with an extracted ion chromatograms at *m/z* = 1449

First, the purified compound **3A** was incubated in phosphate buffer at pH 7.4 and analyzed with RP-HPLC coupled to a mass spectrometer over 12 h. An obvious decrease of **3A** with a half-life time of approximately 2 h was observed with a simultaneous rise of **3B** (Fig. 1a). The extracted ion chromatograms (EIC) clearly confirmed this conversion and revealed that both peaks possess the same molecular mass of *m/z* = 1449 (Fig. 1b).

Interestingly, when the reaction of C-MiniPEG-LARLLT and **1** was performed in acetate buffer at pH 5, the coupling still took place. However, the product **3A** was “stable” and no conversion was observed (see Figure S1A). Furthermore, when the isolated secondary species **3B** was dissolved in 100 mM citric acid (pH ~2.0), no conversion back to **3A** was detected (see Figure S1B).

The same experiments were performed for the oxaliplatin precursor **2** and C-MiniPEG-LARLLT as well as the shuffled analog C-MiniPEG-RTALLL. The LC–MS measurements revealed also for these compounds (**4** and **5**) a distinct conversion comparable to the cisplatin analog **3** (see Figure S2). Again, this phenomenon did not take place in acetate buffer at pH 5 and the secondary species (**4B** and **5B**) could not be converted back to the primary compounds **4A** and **5A** by lowering the pH. A stability test of the purified **4B** in phosphate buffer (pH 7.4) for 24 h revealed no further conversions, but a very slow degradation of about 0.5%/h.

**Scheme 3:**
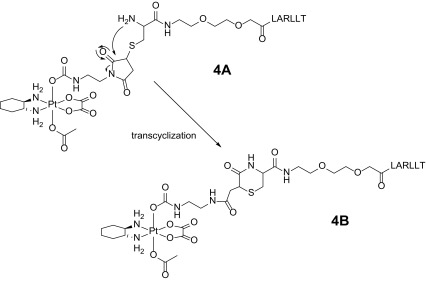
Schematic illustration of the irreversible transcyclization reaction of** 4A** leading to the secondary species** 4B**

To exclude the involvement of different functional moieties of the peptide sequence in the conversion process, three additional peptides were investigated (see Figure S3). In case of the first peptide (C-MiniPEG-LAGLLT), the arginine was exchanged by a glycine to exclude any possible effects of the guanidine moiety. The second peptide was amidated to remove the possibility of reactions of the free terminal carboxylic acid and the third peptide possesses a GGG linker instead of the MiniPEG moiety. However, all three peptides showed the same kind of conversion within the first few hours after incubation with **2** in phosphate buffer (pH 7.4) (see Figure S4). Thus, on the basis of these results, we could rule out that one of the three functional building blocks of the peptide is responsible for the conversion phenomenon. Further HPLC–MS incubation experiments with the peptides C-MiniPEG-LARLLT and C-MiniPEG-RTALLL alone in phosphate buffer at pH 7.4 revealed that no conversion takes place. Solely, the oxidation of the thiols to the disulfide species could be clearly observed with a half-life time of around 12 h (see Figure S5). Also ^195^Pt-NMR measurements of **4A** and **4B** showed nearly identical shifts for both compounds. Therefore, as the LARLLT/RTALLL peptides alone showed no conversion and the platinum core seems not to be affected, only the area of the maleimide moiety remains as reaction site. Thus, most likely a nucleophilic attack of the cysteine amino group on the maleimide carbonyl takes place (see Scheme 3, conversion of **3A** to **3B** see Scheme S1). Although a complete characterization of the conjugates by 2D-NMR spectroscopy due to the very large/complex structures was not possible, a comparison of the 2D-NMR spectra of compound **4A** and **4B** in DMF-*d*_7_ clearly supports this assumption. Especially the occurrence of an additional NH-signal in the ^15^N-^1^H-HSQC spectrum and a coupling of the OC(=O)NH proton to this NH in the TOCSY spectrum confirmed this reaction. Notably, such a transcyclization reaction between cysteines coupled to maleimides was already reported, but only in few examples in literature [34–36].

Due to the fact that the platinum core is still intact and also the LARLLT/RTALLL peptide sequence is completely unaffected, this intramolecular rearrangement reaction should not influence the biological activity. On the contrary, the transcyclization possibly prevents a retro-Michael reaction [37] of the maleimide moiety with thiol-containing molecules.

### Reduction experiments

The actual active species of these peptide-targeted platinum(IV) prodrugs are their respective platinum(II) complexes. Therefore, the reduction of the platinum(IV) moiety with release of the axial targeting ligand and the active platinum(II) compounds is crucial. Nevertheless, the reduction rate should not be too high, as the compounds need sufficient time to accumulate at the tumor site.

**Fig. 2:**
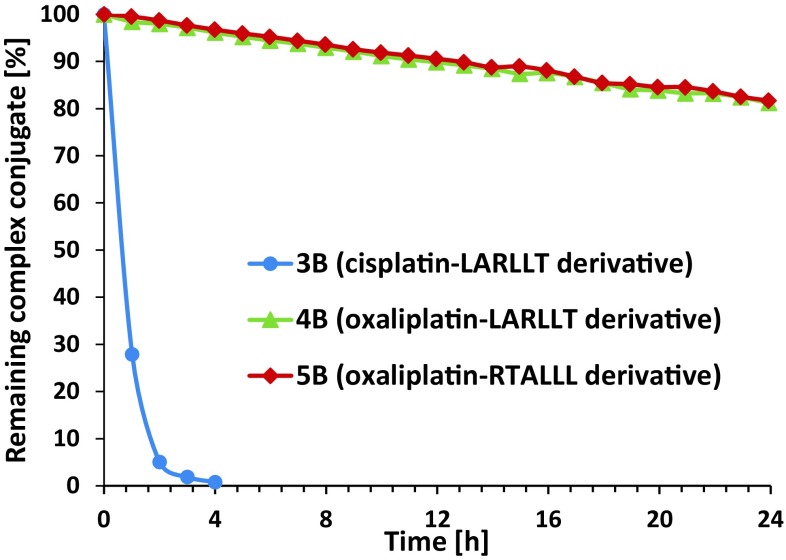
Reduction of compounds** 3B**–**5B** with 10 eq. ascorbic acid in phosphate buffer at pH 7.4 monitored by RP-HPLC

All three complexes were dissolved in 100 mM phosphate buffer (pH 7.4) and after addition of 10 eq. of ascorbic acid the decrease of the compound peak was monitored by RP-HPLC (the stable transcyclization products were used). The experiment showed that the cisplatin-containing conjugate **3B** was reduced much faster in comparison to the two oxaliplatin analogs **4B** and **5B**. In general, this result is not unexpected, as the chloride ligands of the cisplatin-based compound are known to facilitate the electron transfer [38]. The almost identical results of the two oxaliplatin conjugates can be explained, as the only difference is the sequence of the amino acids. Overall, these results are in good accordance to previous experiments with similar platinum cores [33] (Fig. 2).

### Investigations of the platinum(IV) conjugates in cell culture

**Fig. 3:**
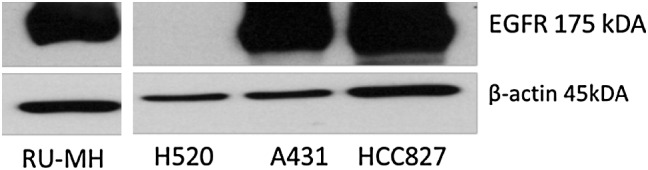
EGFR protein level of selected cell lines. Membrane-enriched fractions of the indicated cell lines were resolved by gel electrophoresis and EGFR expression detected via Western blot. β-Actin was used as a loading control

In order to allow the biological testing of the novel derivatives, a cell line panel was selected based on EGFR expression levels and sensitivity to EGFR-inhibitory treatment (Table 1; Fig. 3). Consequently, A431 as well as RUMH cells were used due to their distinct overexpression of the wild-type EGFR (EGFR/wt) and moderate sensitivity to EGFR inhibition in the µM range. In addition, HCC827 cells were included; a cell model known for its amplification of the mutated EGFR version (E746-A750 del), which renders these cells hypersensitive to EGFR inhibition. Finally, H520 cells were chosen as a negative control for this study as they lack EGFR expression and, hence, do not respond to treatment with EGFR inhibitors.

**Table 1:** IC_50_ values of gefitinib and erlotinib in our selected cell line panel after 72 h of treatment

Cell line	EGFR expression	Gefitinib (µM)	Erlotinib (µM)
		IC_50_ ± SD	IC_50_ ± SD
A431	EGFR/wt overexpression	14.1 ± 0.98	7.6 ± 1.7
RUMH	EGFR/wt overexpression	7.5 ± 0.51	23.0 ± 2.6
HCC827	Overexpression of EGFR with the sensitizing mutation (E746-A750 del)	0.06 ± 0.02	0.06 ± 0.00
H520	No EGFR expression	>25	>25

**Fig. 4:**
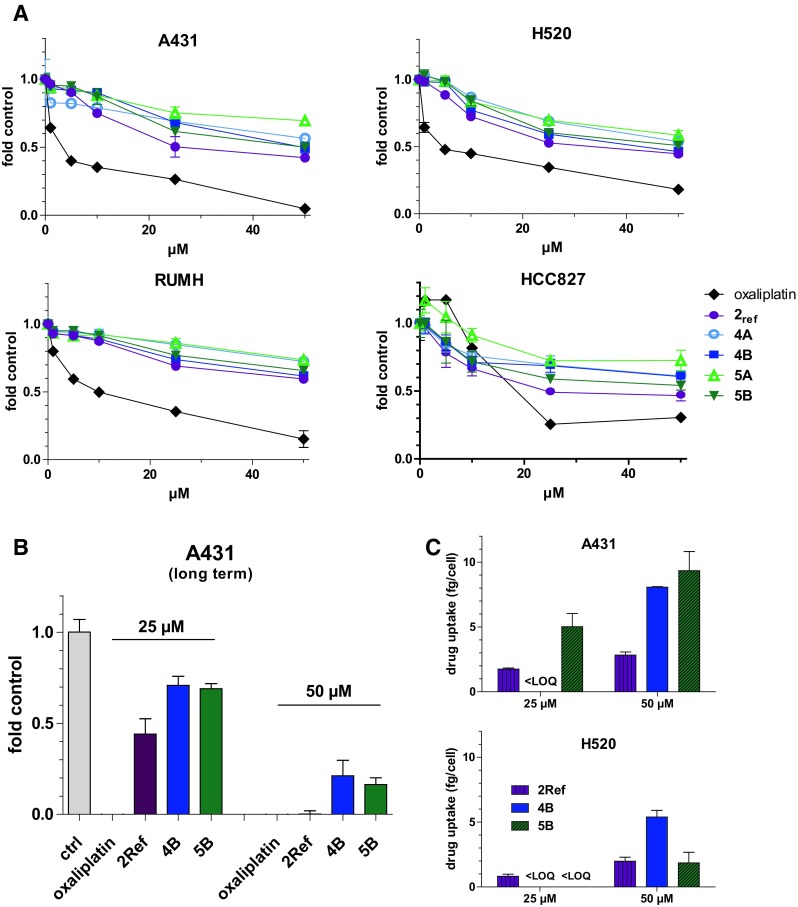
Biological activity of targeted peptide-containing platinum drugs. **a** Cytotoxicity of indicated platinum drugs in A431, H520, RUMH and HCC827 cells after 72 h treatment. Viability was determined using MTT assay. The values given are the mean ± the standard deviation of triplicates from one representative experiment out of three, yielding similar results. **b** Effect of long-term treatment (14 days) of the indicated platinum drugs (25 and 50 µM) on A431 cells. The values given are the mean ± the standard deviation of three experiments performed in duplicates normalized to untreated cells (ctrl) (values for oxaliplatin are too low to be seen in this graph). **c** Cellular uptake (3 h) of indicated platinum drugs (25 and 50 µM) in A431 and H520 cells measured by ICP-MS. Treated wells without cells were used as blanks. The values given are the mean ± the standard deviation of one experiment performed in triplicates. *LOQ* limit of quantification

As a first step to evaluate our new compounds, the impact on cell viability after 72 h treatment was investigated by MTT assay (Fig. 4a) in comparison to the succinimide reference compound of **2** (unable to bind to thiol groups, denoted as **2**_**ref**_). Comparable to other platinum(IV) complexes [39, 40], this reference complex **2**_**ref**_ was distinctly less active than oxaliplatin, which is based on the prodrug nature of the compound. However, unexpectedly, the peptide-coupled oxaliplatin derivatives were even less active than **2**_**ref**_ and no difference in the activity was observed with regard to the EGFR expression level or whether LARLLT or RTALLL was attached to the platinum(IV) complex (as assumed above, also no significant differences between the A and B derivatives were observed). Furthermore, also in case of the cisplatin analog **3**, no correlation between anticancer activity and EGFR status was found (data not shown). In order to investigate whether a longer drug exposure time is needed for prodrug activation, long-term clonogenic assays with 14 days of drug treatment (25 and 50 µM) were performed in EGFR-overexpressing A431 cells (Fig. 4b). However, although the activity of all drugs was distinctly increased, again no differences between the LARLLT and RTALLL conjugates were found. In line with these data, ICP-MS uptake studies showed no correlation between drug accumulation and the EGFR status of the tested cell lines (Fig. 4c). Notably, also other authors recently reported about difficulties to correlate the cytotoxicity and cell uptake with the receptor expression in case of RGD (arginine-glycine-aspartate) peptide-containing platinum complexes targeting the α_v_β_3_ integrin receptor [41].

### Accumulation studies using FITC-labeled peptides

**Scheme 4:**
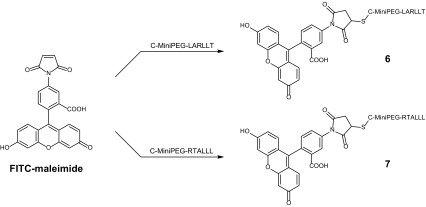
Reaction scheme for the synthesis of the EGFR peptide-coupled FITC derivatives** 6** and** 7**

As the investigated LARLLT-conjugated platinum compounds unexpectedly did not show any EGFR-specific drug accumulation nor anticancer activity, fluorophore-coupled LARLLT/RTALLL peptides were synthesized to re-evaluate the general targeting ability of the peptide sequences. Commercially available maleimide-functionalized fluorescein (in general abbreviated as its isothiocyanate form, FITC) was coupled to C-MiniPEG-LARLLT and C-MiniPEG-RTALLL yielding derivatives **6** and **7**, respectively, after purification (Scheme 4).

**Fig. 5:**
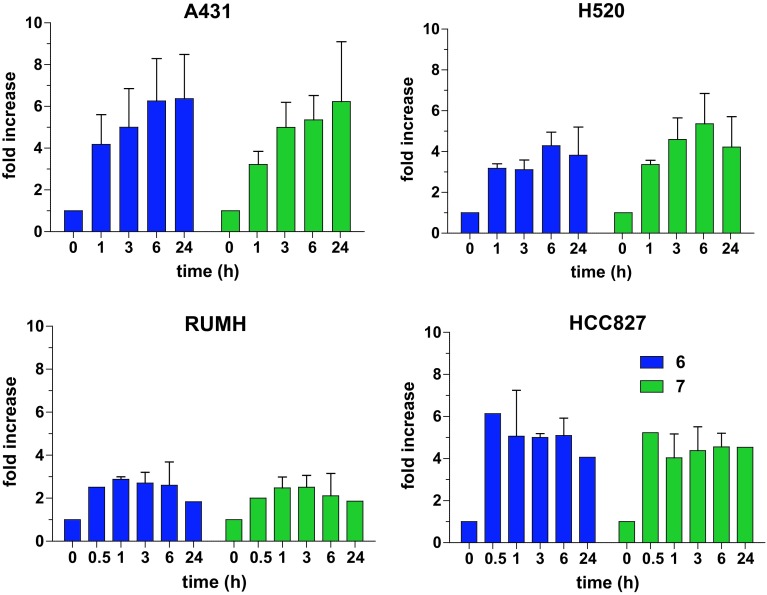
Uptake of the FITC-labeled LARLLT/RTALLL peptide sequence in different cell lines. Cells were treated with either 10 µM **6** or **7** for the indicated time periods. Increase in fluorescence compared to untreated cells was measured by flow cytometry. The values given are the mean ± the standard deviation of two independent experiments

The impact of the EGFR expression levels on the uptake of the FITC-labeled peptides **6** and **7** was then tested by flow cytometry after several incubation times (Fig. 5). Again, the panel of cell lines with different EGFR status was used. Comparable to the conjugated platinum drugs, also with these compounds, no EGFR dependency was found either in total uptake or in uptake kinetic. In addition, at no point in time in none of the tested cell models, cellular accumulation of LARLLT was superior to the shuffled RTALLL reference.

Notably, in most of the publications in literature, LARLLT was used as a targeting peptide without a shuffled control [27, 42, 43]. This makes an evaluation quite difficult as, for example, our uptake studies (Fig. 4c), when not considering the RTALLL data, would also suggest a significantly increased accumulation into the EGFR-expressing A431 cell line. Some promising data were reported for nanoformulations like liposomes [25] and micelles [26], which harbor a huge number of LARLLT peptides attached to their surface. However, also in case of LARLLT-containing silica nanoparticles no correlation of drug uptake with the EGFR status (even when the EGFR was genetically silenced in a cancer cell line) was found. However, coupling of EGFR-specific single-domain antibodies (sdAbs) to the same nanoparticles resulted in highly specific uptake. This implies that not the nanoformulation in general but the LARLLT peptide was responsible for the lack of EGFR specificity [44]. The authors of this study mentioned that the chemical nature of the conjugates and characteristics such as charge and polarity may have a substantial influence on the tumor-targeting abilities of LARLLT, which is supported by our study.

## Conclusion

In this study, we successfully synthesized the first EGFR-targeting, peptide-coupled platinum(IV) conjugates. Thereto, maleimide-functionalized platinum(IV) complexes were attached to the literature-known EGFR-affine peptide LARLLT in comparison to the shuffled RTALLL analog. Subsequently, the EGFR-dependent anticancer activity and their targeting properties were evaluated in different cell lines. However, these studies revealed that the activity of the targeted complexes did not correlate with the EGFR status. Furthermore, cell uptake studies showed no EGFR specificity compared to the reference complex. Thus, proof-of-principle studies using fluorescein-labeled LARLLT and RTALLL were conducted to re-evaluate the targeting ability of the peptide. In line, these data suggested that LARLLT (when compared to RTALLL) is not a suitable targeting moiety to improve the specificity of such small-molecule compounds for EGFR-overexpressing cells. As a consequence, these results prompt that also for literature-known peptide sequences (1) the targeting ability has to be re-evaluated in an appropriate cell line panel and (2) a shuffled reference of the targeting peptide obligatorily has to be included into the study design to exclude false positive results originating from unspecific interactions.

Independently from the biological evaluation, we were able to identify an interesting transcyclization reaction after coupling of the terminal cysteine to the maleimide moiety. This is of special interest as it is a quite common coupling strategy for the attachment of bioactive compounds to peptides. Interestingly, only few references described this intramolecular reaction so far [34–36], although it should frequently occur in this type of coupling strategy. Ongoing studies will clarify if such transcyclization processes could prevent a possible retro-Michael reaction of the maleimide moiety with thiol-containing molecules and, therefore, increase the stability of such peptide-coupled drugs in biological matrices such as blood serum.

## Experimental part

### Materials and methods

Potassium tetrachloridoplatinate (K_2_PtCl_4_) was purchased from Johnson Matthey (Switzerland). Water used for synthesis was taken from a reverse osmosis system and distilled twice. For HPLC measurements Milli-Q water (18.2 MΩ cm, Merck Milli-Q Advantage, Darmstadt, Germany) was used. Other chemicals and solvents were purchased from commercial suppliers (Sigma Aldrich, Merck, Acros, Fluka and Fisher Scientific) and used without further purification. The starting platinum(II) compounds cisplatin [29] and oxaliplatin [30] were synthesized according to literature-known procedures. Hydrogen peroxide (50%) was used for oxidation of the complexes in either methanol [31] or acetic acid [32] as a solvent, yielding the unsymmetrically oxidized platinum(IV) precursors. Thereafter, the ligands with the maleimide or succinimide moiety were coupled to yield compounds **1, 2** and **2**_**Ref**_ as recently published [33, 45]. Cys-MiniPEG-LARLLT and Cys-MiniPEG-RTALLL (MiniPEG = 2-(2-(2-aminoethoxy)ethoxy)acetic acid, both TFA salts, >95% purity) were purchased from Biomatik Corporation (Cambridge, Canada). Electrospray ionization (ESI) mass spectra were recorded on a Bruker AmaZon SL ion trap mass spectrometer in positive and/or negative ionization mode by direct infusion. High-resolution mass spectra were measured on a Bruker maXis™ UHR ESI time-of-flight mass spectrometer. All mass spectra were recorded at the Mass Spectrometry Centre of the University of Vienna. One- and two-dimensional ^1^H-, ^13^C-, ^15^N- and ^195^Pt-NMR spectra were recorded on a Bruker Avance III 500 MHz spectrometer at 500.10 (^1^H), 127.75 (^13^C), 50.68 (^15^N), and 107.51 (^195^Pt) MHz at 298 K. For ^1^H- and ^13^C-NMR spectra the solvent residual peak was taken as internal reference, whereas ^195^Pt-shifts were referenced relative to external K_2_PtCl_4_ and ^15^N-shifts relative to external NH_4_Cl. Elemental analysis measurements were performed on a Perkin Elmer 2400 CHN Elemental Analyzer at the Microanalytical Laboratory of the University of Vienna.

### Synthesis and characterization

#### General procedure for the synthesis of peptide-conjugated complexes **3**–**5**

The maleimide-functionalized platinum(IV) compound and the cysteine-containing peptide (0.7–0.8 eq.) were transferred into a vial, phosphate buffer (pH 7.4, ~0.45 mL/µmol platinum complex) was added and the solution was stirred for around 4 h. Both of the compound peaks were purified by preparative RP-HPLC on a Waters XBridge C18 column using H_2_O (0.1% HCOOH) and acetonitrile (ACN) as eluents. The product fractions were collected and thereafter lyophilized. Purity (>95%) was confirmed by analytical RP-HPLC measurements (see Figure S6).

##### Compound **3**

The compound was synthesized from 20 mg (*OC*-6-44)-diamminedichloridomethoxido[2-(2,5-dioxo-2,5-dihydro-1*H*-pyrrol-1-yl)ethylcarbamato]platinum(IV) (**1**, 38.9 µmol) and 39.7 mg C-MiniPEG-LARLLT (0.8 eq., 31.1 µmol). Yield: 16.7 mg (37%) pale-yellow powder; HRMS (ESI-TOF): calcd. for [C_48_H_91_Cl_2_N_15_O_17_PtS-Na^+^H^+^]^2+^: 735.7712, found: 735.7732.

##### Compound **4**

The compound was synthesized from 30 mg (*OC*-6-34)-acetato[(1*R*,2*R*)-cyclohexane-1,2-diamine]oxalato[2-(2,5-dioxo-2,5-dihydro-1*H*-pyrrol-1-yl)ethylcarbamato]platinum(IV) (**2**, 45.6 µmol) and 40.8 mg C-MiniPEG-LARLLT (0.7 eq., 31.9 µmol). Yield: 25.3 mg (50%) white powder; HRMS (ESI-TOF): calcd. for [C_57_H_99_N_15_O_22_PtS-Na^+^H^+^]^2+^: 798.8214, found: 798.8242.

##### Compound **5**

The compound was synthesized from 30 mg (*OC*-6-34)-acetato[(1*R*,2*R*)-cyclohexane-1,2-diamine]oxalato[2-(2,5-dioxo-2,5-dihydro-1*H*-pyrrol-1-yl)ethylcarbamato]platinum(IV) (**2**, 45.6 µmol) and 40.8 mg C-MiniPEG-RTALLL (0.7 eq., 31.9 µmol). Yield: 22.4 mg (43%) white powder; HRMS (ESI-TOF): calcd. for [C_57_H_99_N_15_O_22_PtS-Na^+^H^+^]^2+^: 798.8214, found: 798.8250.

#### General procedure for the synthesis of the fluorescein-peptide conjugates

The cysteine-functionalized peptide was dissolved in phosphate buffer (pH 7) and was added to a solution of FITC-maleimide in DMSO. After the solution was stirred for 1 h the product was purified by preparative RP-HPLC on a Waters XBridge C18 column using H_2_O (0.1% HCOOH) and ACN as eluents. The product fractions were collected and thereafter lyophilized.

##### Compound **6**

The compound was synthesized from 20 mg C-MiniPEG-LARLLT (15.7 µmol) in 8 mL phosphate buffer (pH 7) and 6.7 mg FITC-maleimide (1 eq., 15.7 µmol) in 1.3 mL DMSO. Yield: 16.0 mg (75%) yellow powder; ESI-MS: calcd. for [C_64_H_88_N_12_O_19_S-H^+^]^+^: 1361.61, found: 1361.91.

##### Compound **7**

The compound was synthesized from 20 mg C-MiniPEG-RTALLL (15.7 µmol) in 8 mL phosphate buffer (pH 7) and 6.7 mg FITC-maleimide (1 eq., 15.7 µmol) in 1.3 mL DMSO. Yield: 16.6 mg (78%) yellow powder; ESI-MS: calcd. for [C_64_H_88_N_12_O_19_S-H^+^]^+^: 1361.61, found: 1361.72.

### RP-HPLC studies

The RP-HPLC measurements for monitoring the coupling reaction and the conversion were either performed on a Dionex Summit System equipped with a Waters XBridge BEH C18 column (130 Å, 5 µm, 4.6 × 150 mm) or on a Thermo Scientific Dionex Ultimate 3000 Rapid Separation LC system equipped with a Waters Acquity UPLC BEH C18 column (130 Å, 1.7 µm, 3.0 × 50 mm). Milli-Q water, containing 0.1% formic acid, and methanol or ACN were used as eluents and as a standard method, a gradient from 5 to 95% of the organic solvent was used. The samples were dissolved in phosphate buffer (pH 7.4) and were incubated in the autosampler at 20 °C. The column compartment was temperature controlled at 25 °C.

All the LC–MS measurements were performed on an Agilent 1260 Infinity system using either a Waters Atlantis T3 C18 column (100 Å, 3 µm, 2.1 × 150 mm) or a Waters Acquity UPLC BEH C18 column (130 Å, 1.7 µm, 3.0 × 50 mm). Milli-Q water and acetonitrile, both containing 0.1% formic acid, were used as eluents and a gradient from 5 to 95% of the acetonitrile solution over 15 min with 0.2 mL/min was used. The samples were dissolved in phosphate buffer (pH 7.4) and were incubated in the autosampler at 20 °C. The mass spectra were recorded on a Bruker AmaZon SL electrospray ionization ion trap mass spectrometry system in positive ionization mode using a drying gas flow of 10 L/min (350 °C), a nebulizer pressure of 35 psi and a capillary voltage of 4000 V. HyStar 3.2 and Data Analysis 4.0 software package (Bruker Daltonics) were used for instrument control and data evaluation.

### RP-HPLC reduction experiments

The reduction studies were performed on a Thermo Scientific Dionex Ultimate 3000 Rapid Separation LC system equipped with a Waters Acquity UPLC BEH C18 column (130 Å, 1.7 µm, 3.0 × 50 mm). Milli-Q water and acetonitrile, both containing 0.1% formic acid, were used as eluents and as a standard screening gradient, a gradient from 5 to 95% of the organic eluent over 10 min with 0.6 mL/min was carried out. The samples and 10 eq. of ascorbic acid were dissolved in phosphate buffer (pH 7.4) and were incubated in the autosampler at 20 °C. The column compartment was temperature controlled at 25 °C.

### Cell culture

The following human cell models were used in this study: the renal cell carcinomas RUMH, the squamous cell carcinoma H520, the epidermoid carcinoma A431, as well as the non-small-cell lung cancer (NSCLC) line HCC827 (sources and used medium are summarized in (Table 2). Unless otherwise indicated, the cells were cultivated in humidified incubators (37 °C, 21% O_2_, 5% CO_2_) in full culture medium, containing 10% fetal calf serum (PAA, Linz, Austria). Cell cultures were periodically checked for *Mycoplasma* contamination.

**Table 2:** Detailed information on the used cell lines

Cell line	Characteristics	Growth medium	Source
A431	EGFR wild-type overexpression, erlotinib-sensitive	RPMI-1640	ATCC
HCC827	Erlotinib-sensitive due to the EGFR mutation (delE746-A750)	RPMI-1640	ATCC
RUMH	EGFR wild-type overexpression, erlotinib-sensitive	RPMI-1640	Established at the ICR
H520	No EGFR expression, erlotinib-resistant	RPMI-1640	ATCC

*ATCC* American Type Culture Collection Manassas VA, *ICR* Institute of Cancer Research, Vienna

### Cytotoxicity assay

Cells were plated (2 × 10^3^ cells/well) in 96-well plates and allowed to recover for 24 h. Subsequently, the dissolved drugs were added. After 72 h drug exposure, the proportion of viable cells was determined by MTT assay following the manufacturer’s recommendations (EZ4U, Biomedica, Vienna, Austria). Cytotoxicity was expressed as IC_50_ values calculated from full dose–response curves using GraphPad Prism software. For long-term exposure, A431 cells (200 cells/well) were seeded in 24-well plates and allowed to recover for 24 h. Then, the cells were exposed to the indicated drugs (25 or 50 µM) for 14 days. After washing with phosphate-buffered saline (PBS), the cells were fixed with methanol (−20 °C, 20 min) and after another washing step stained with crystal violet (1 h). The washed and dried plates were then measured for fluorescence (with 633 nm excitation and 610/30 nm bandpass emission filter) with the imager Typhoon Trio (GE Healthcare Life Sciences). The sum of fluorescence intensities per well was measured with ImageJ and, after blank subtraction, normalized to the untreated cells.

### Western blot analysis

To assess the EGFR expression levels, membrane-enriched fractions of untreated cells cultivated under normal cell culture conditions were prepared and resolved by SDS-PAGE and transferred onto a polyvinylidene difluoride membrane for Western blotting as previously described [46]. The following antibodies were used: EGFR (monoclonal rabbit, dilution 1:1000 from Cell Signaling) and β-actin (monoclonal mouse, dilution 1:5000 from Sigma Aldrich). Additionally, horseradish peroxidase-labeled secondary antibodies from Santa Cruz Biotechnology were used at working dilutions of 1:10,000.

### Cellular drug uptake

Cells (3 × 10^5^ cells/well) were seeded in 6-well plates and allowed to recover for 24 h. Then, cells were exposed to the drugs with the indicated concentrations for 3 h. Subsequently, the cells were washed three times with 2 mL PBS and platinum was extracted by incubating the cells with 500 µL HNO_3_ (HPLC grade) for 1 h. From the suspension aliquots of 400 µL were diluted 20-fold in dd H_2_O. The experiment was performed in triplicates. Cell-free wells exposed to the according drugs were used as blanks. Cells from three additional wells were trypsinized and counted to determine the cell number per well.

### Flow cytometry

Cells were seeded in 6-well plates at a concentration of 4 × 10^5^ cells per well in 2 mL growth media containing 10% FCS. Then, cells were incubated over night at 37 °C and 5% CO_2_. The growth media was removed and the adherent cells were washed with PBS two times before further treatment. As a next step, the drugs were added in serum-free media obtaining a final concentration of 10 µM followed by incubation at 37 °C. At the indicated points in time, cells were washed two times with PBS and harvested by trypsinization. After centrifugation at 2000 rpm for 3 min, the pellet was resuspended in 500 µL FACS-PBS (7.81 mM Na_2_HPO_4_ × 2H_2_O, 1.47 mM KH_2_PO_4_, 2.68 mM KCl and 0.137 M NaCl) and transferred into FACS-tubes. Fluorescence intensities of 10,000 cells per sample were measured using a FACS Calibur (Becton–Dickinson, Palo Alto, CA). The results were analyzed and quantified using Cell Quest Pro software.

### ICP-MS measurements

Milli-Q water (18.2 MΩ cm, Milli-Q Advantage, Darmstadt, Germany) was used for all dilutions for ICP-MS measurements. Nitric acid (≥69%, p.a., Fluka, Buchs, Switzerland) was used without further purification. Platinum and rhenium standards for ICP-MS measurements were derived from CPI International (Amsterdam, The Netherlands). All other reagents and solvents were obtained from commercial sources and were used without further purification.

The ICP-MS Agilent 7500ce (Agilent Technologies, Waldbronn, Germany) was equipped with a CETAC ASX-520 autosampler (Nebraska, USA) and a MicroMist nebulizer at a sample uptake rate of approx. 0.25 mL/min. The Agilent MassHunter software package (Workstation Software, version B.01.01, Build 123.11, Patch 4, 2012) was used for data processing. The experimental parameters for ICP-MS are summarized in Table 3. The instrument was tuned on a daily basis to achieve maximum sensitivity.

**Table 3:** Parameters of the Agilent 7500ce ICP-MS

RF power (W)	1560
Cone material	Nickel
Carrier gas (L/min)	0.8–1.0
Make up gas (L/min)	0.1–0.3
Plasma gas (L/min)	15
Monitored isotopes	^185^Re, ^194^Pt, ^195^Pt
Dwell time (s)	0.3
Number of replicates	10
Number of sweeps	100

The limit of quantification (LOQ) was determined by the formula ${\text{LOQ}} = \bar{x} + 10 \times s$ with $\bar{x}$ as the average sample blank value and $s$ as the standard deviation of x (*n* = 7)

## Electronic supplementary material

Below is the link to the electronic supplementary material.

Supplementary material 1 (PDF 405 kb)
