# Supplementary material for: EGFR-targeting peptide-coupled platinum(IV) complexes
Source: J Biol Inorg Chem. 2017 Apr 12;22(4):591–603. doi: 10.1007/s00775-017-1450-7 (PMC5443859; doi:10.1007/s00775-017-1450-7)
Supplement: Supplementary file 1 — Supplementary material 1 (PDF 405 kb) [file 775_2017_1450_MOESM1_ESM.pdf]

Electronic Supplementary Information

EGFR-targeting peptide-coupled platinum(IV) complexes

Josef Mayr[a], Sonja Hager[b], Bettina Koblmüller[b], Matthias H. M. Klose[a], Britta Fischer[a], Karla Pelivan[a], Walter Berger[bc], Petra Heffeter*[bc], Christian R. Kowol*[ac], Bernhard K. Keppler[ac]

[a] University of Vienna, Institute of Inorganic Chemistry, Waehringer Strasse 42, A-1090, Vienna, Austria.
[b] Institute of Cancer Research and Comprehensive Cancer Center, Medical University of Vienna, Borschkegasse 8a, A-1090, Vienna, Austria.
[c] Research Platform ''Translational Cancer Therapy Research'' University of Vienna, Waehringer Strasse 42, A-1090, Vienna, Austria.

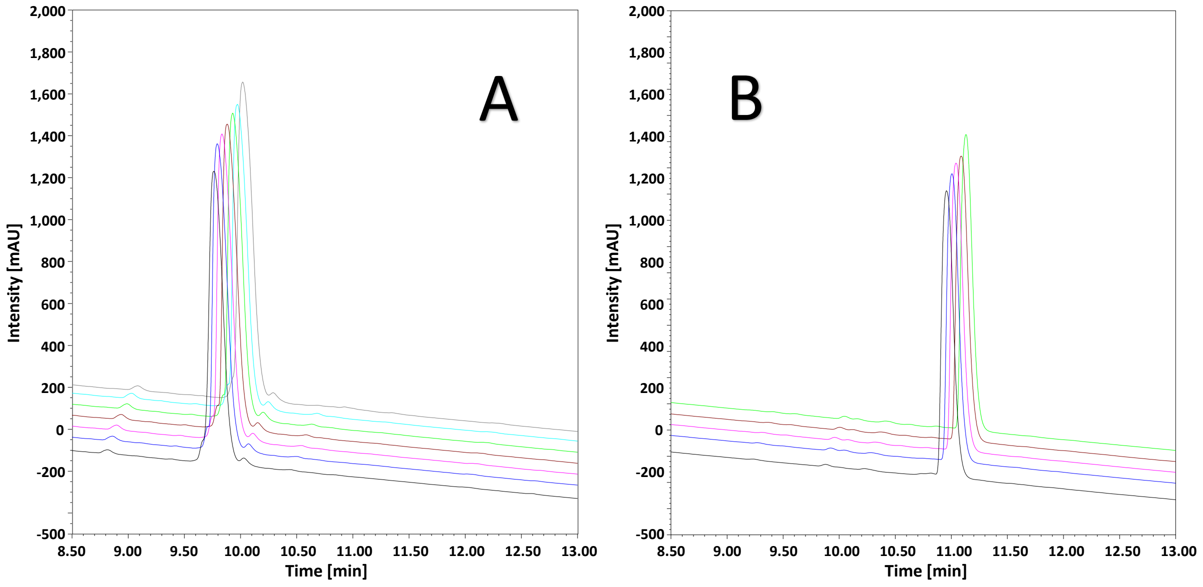

Figure S1: A) Reaction of compound **1** and C-MiniPEG-LARLLT in 50 mM acetate buffer (pH=5) to **3A** after 0, 0.5, 1, 1.5, 2, 2.5 and 24 hours and B) stability of **3B** in 100 mM citric acid (pH=2) after 0, 2, 4, 6, 8 h and 4 days (from front to back).

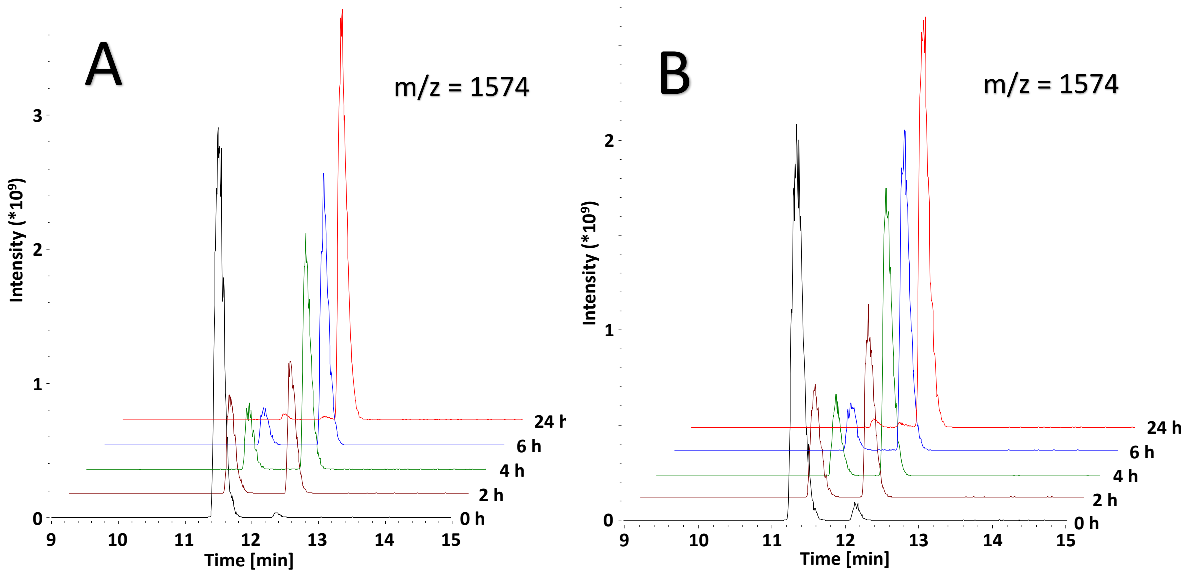

Figure S2: Conversion reaction after coupling the compound **2** to (A) C-MiniPEG-LARLLT or (B) C-MiniPEG-RTALLL in phosphate buffer (pH=7.4) monitored by LC-MS with the corresponding extracted ion chromatogram.

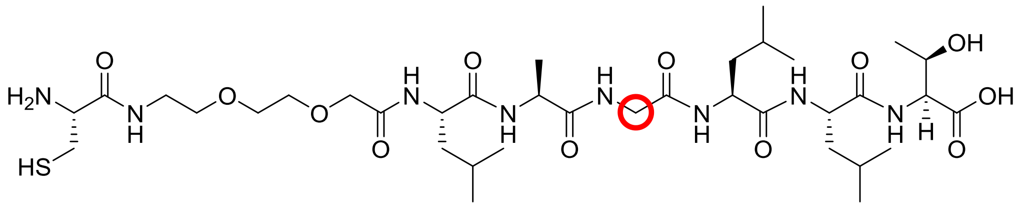

C-MiniPEG-LAGLLT

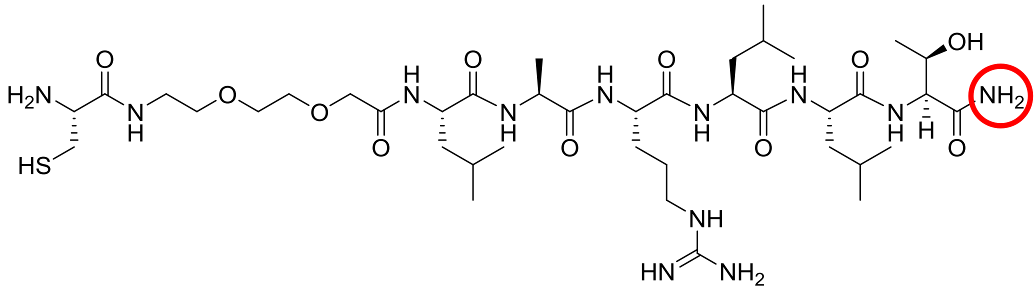

C-MiniPEG-LARLLT-NH_2

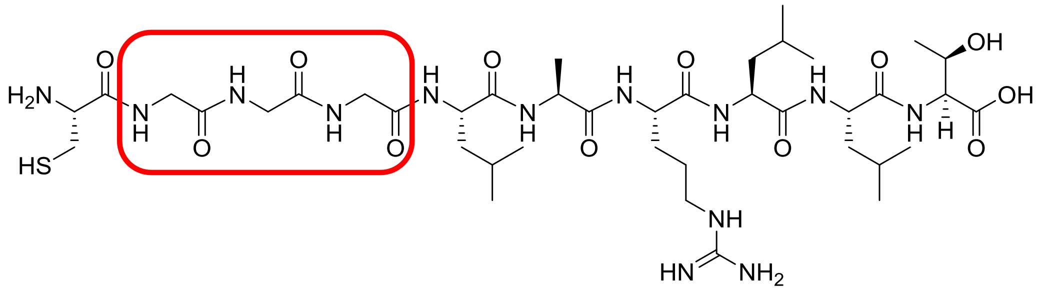

C-GGG-LARLLT

Figure S3: Specifically modified peptides used for evaluation of the conversion phenomenon.

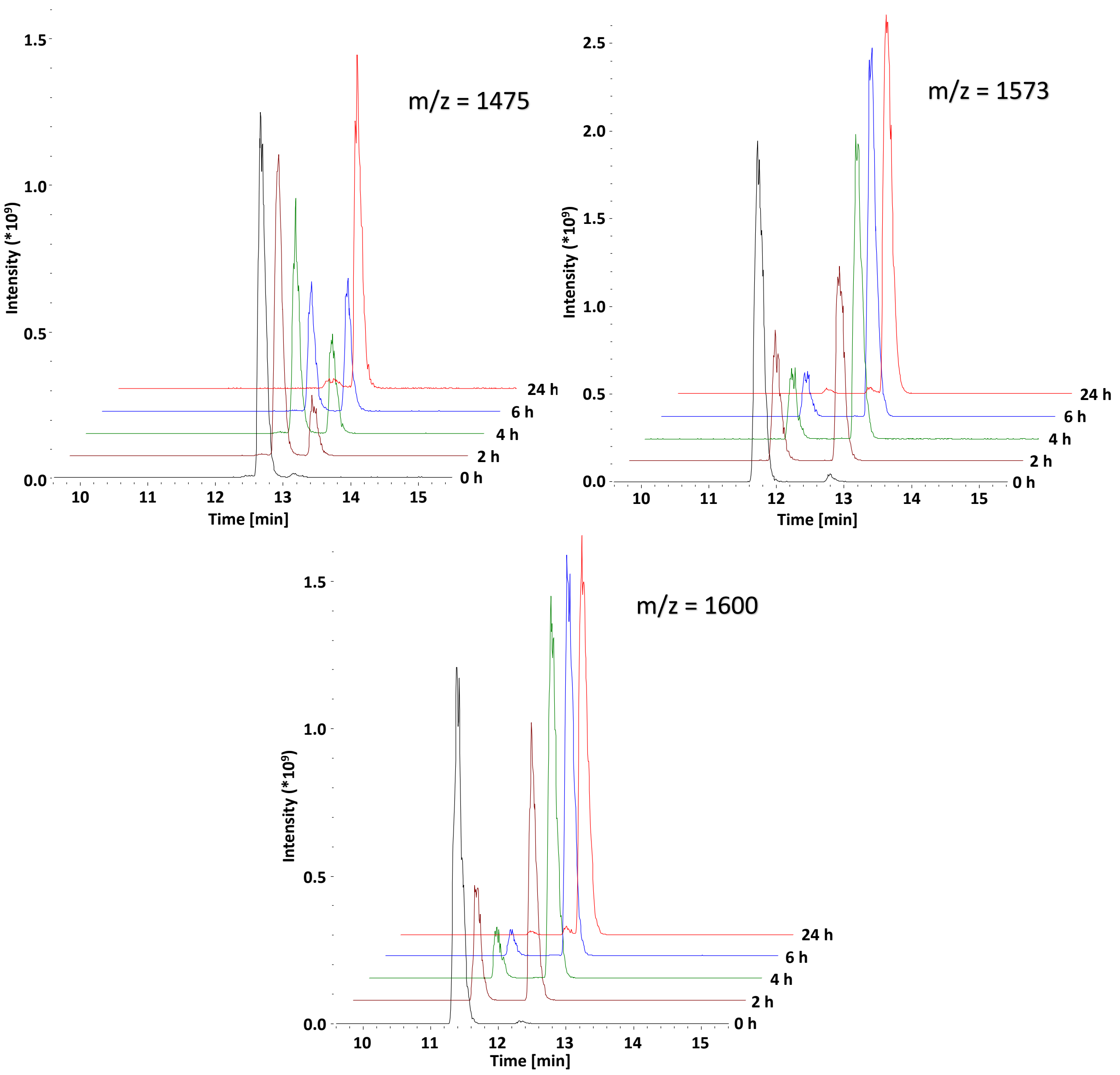

Figure S4: Conversion reaction after coupling the compound **2** to (A) C-MiniPEG-LAGLLT or (B) C-MiniPEG-LARLLT-NH_2 or (C) C-GGG-LARLLT in phosphate buffer (pH=7.4) monitored by LC-MS with the corresponding extracted ion chromatogram.

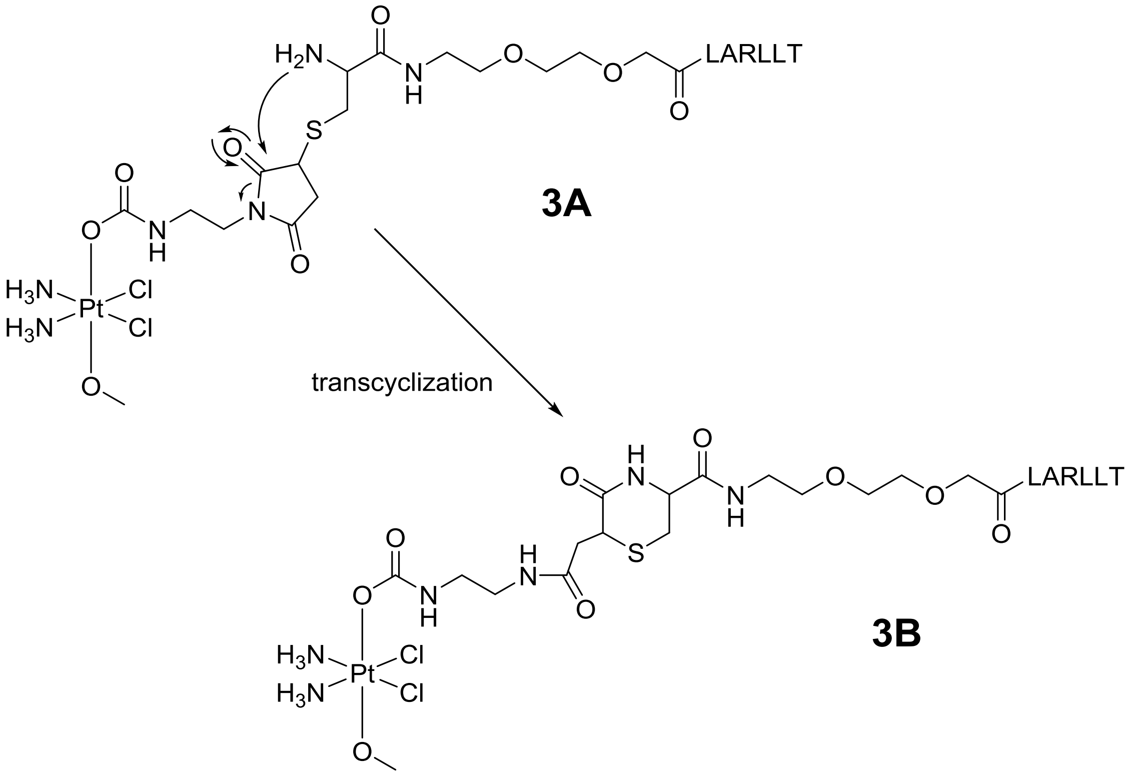

Scheme S1: Schematic illustration of the irreversible transcyclization reaction of **3A** leading to the secondary species **3B**

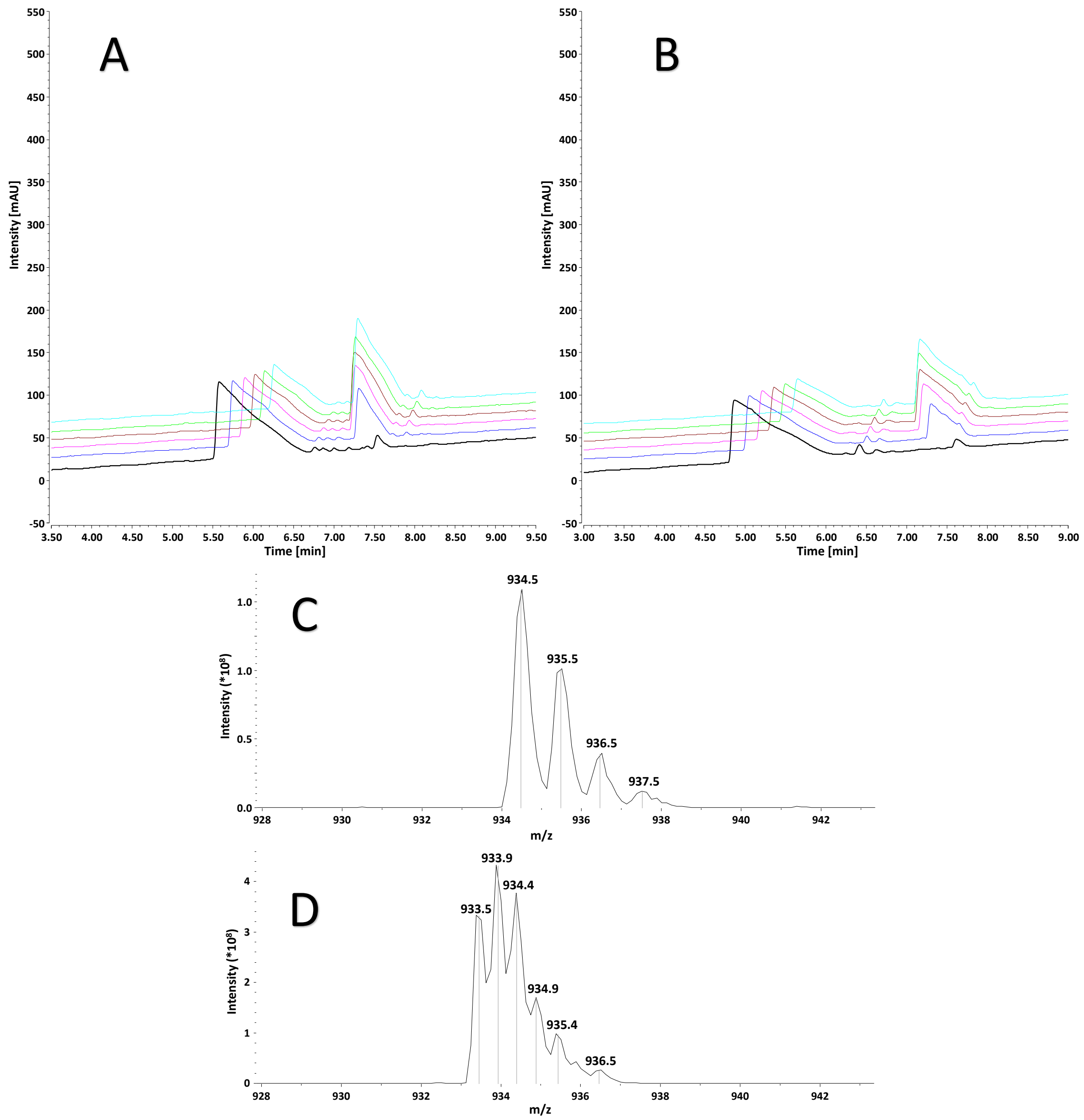

Figure S5: Oxidation of the thiol-functionalized peptides (A) C-MiniPEG-LARLLT and (B) C-MiniPEG-RTALLL to their corresponding disulfides monitored by RP-HPLC and UV/VIS detector at 225 nm and evaluation of the (C) single, positively charged peptide (C-MiniPEG-LARLLT) and the (D) double, positively charged disulfide species of C-MiniPEG-LARLLT by mass spectrometry.

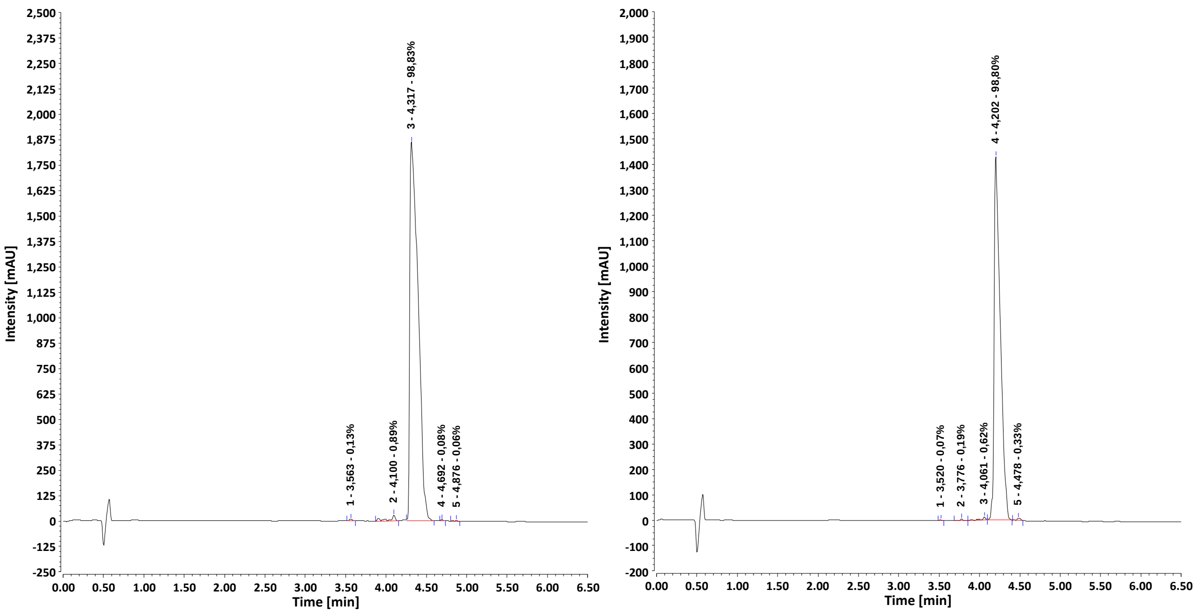

Figure S6: Evaluation of the purity of compounds **4** and **5** by RP-HPLC and UV/VIS detection at 225nm.
